# Toward Ideal Low‐Frequency Noise in Monolayer CVD MoS_2_ FETs: Influence of van der Waals Junctions and Sulfur Vacancy Management

**DOI:** 10.1002/advs.202307196

**Published:** 2024-05-21

**Authors:** Wonjun Shin, Junsung Byeon, Ryun‐Han Koo, Jungmoon Lim, Jung Hyeon Kang, A‐Rang Jang, Jong‐Ho Lee, Jae‐Joon Kim, SeungNam Cha, Sangyeon Pak, Sung‐Tae Lee

**Affiliations:** ^1^ Inter‐University Semiconductor Research Center Department of Electrical and Computer Engineering Seoul National University Seoul 08826 Republic of Korea; ^2^ Department of Semiconductor Convergence Engineering Sungkyunkwan University Gyeonggi‐do Suwon 16419 Republic of Korea; ^3^ Department of Physics Sungkyunkwan University Suwon Gyeonggi‐do 16419 Republic of Korea; ^4^ Division of Electrical Electronic and Control Engineering Kongju National University Cheonan 31080 Republic of Korea; ^5^ Ministry of Science and ICT Sejong 30109 Republic of Korea; ^6^ School of Electronic and Electrical Engineering Hongik University Seoul 04066 Republic of Korea

**Keywords:** 1/f noise, 2D TMDC, contact resistance, copper sulfide, low‐frequency noise, self‐healing effect

## Abstract

The pursuit of sub‐1‐nm field‐effect transistor (FET) channels within 3D semiconducting crystals faces challenges due to diminished gate electrostatics and increased charge carrier scattering. 2D semiconductors, exemplified by transition metal dichalcogenides, provide a promising alternative. However, the non‐idealities, such as excess low‐frequency noise (LFN) in 2D FETs, present substantial hurdles to their realization and commercialization. In this study, ideal LFN characteristics in monolayer MoS_2_ FETs are attained by engineering the metal‐2D semiconductor contact and the subgap density of states (DOS). By probing non‐ideal contact resistance effects using CuS and Au electrodes, it is uncovered that excess contact noise in the high drain current (*I*_D_) region can be substantially reduced by forming a van der Waals junction with CuS electrodes. Furthermore, thermal annealing effectively mitigates sulfur vacancy‐induced subgap density of states (DOS), diminishing excess noise in the low *I*_D_ region. Through meticulous optimization of metal‐2D semiconductor contacts and subgap DOS, alignment of 1/*f* noise with the pure carrier number fluctuation model is achieved, ultimately achieving the sought‐after ideal LFN behavior in monolayer MoS_2_ FETs. This study underscores the necessity of refining excess noise, heralding improved performance and reliability of 2D electronic devices.

## Graphical abstract

An ideal low‐frequency noise characteristic is achieved in MoS_2_ field‐effect transistors (FETs) via optimization of contact electrode and thermal annealing process. The optimized MoS_2_ FETs exhibit ideal 1/f noise behavior in both frequency and drain current domains.

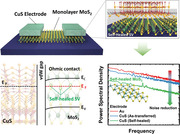

## 1 Introduction

The relentless scaling of silicon complementary metal–oxide–semiconductor technology has successfully reached sub‐10‐nm nodes. However, further scaling has become challenging due to the gate electrostatics of the devices.^[^ ^1^, ^2^ ^]^ To maintain the desired performance, an aggressive reduction in channel thickness is necessary. The ultimate goal is to achieve a field‐effect transistor (FET) channel thickness in the sub‐1‐nm range. However, achieving such thickness in 3D semiconducting crystals poses difficulties due to increased charge carrier scattering at the interfaces between the channel and dielectric, leading to significant mobility degradation.^[^ ^3^ ^]^ A potential solution lies in 2D semiconductors, which have a thickness of ≈0.7 nm in their monolayer form.^[^ ^4^, ^5^, ^6^ ^]^ One example of these materials is transition metal dichalcogenides with the general formula MX_2_, where M represents a transition metal (such as Mo or W), and X represents a chalcogen (such as S, Se, or Te).^[^ ^7^, ^8^, ^9^ ^]^ These 2D semiconductors offer the advantage of having no dangling bonds, which could result in improved interfaces between the channel and dielectric. Early studies using mechanically exfoliated single‐crystalline 2D flakes, as well as recent advancements in large‐area synthesis techniques for 2D monolayers, have showcased the promising characteristics of 2D FETs.^[^ ^10^ ^]^ However, several significant challenges still remain and must be overcome before the full potential of incorporating 2D FETs into future very large‐scale integration technologies can be realized. Specifically, in 2D FETs, diverse forms of excess noise manifest within the low‐frequency spectrum.^[^ ^11^, ^12^, ^13^, ^14^, ^15^, ^16^, ^17^, ^18^, ^19^, ^20^, ^21^, ^22^, ^23^, ^24^, ^25^ ^]^ This substantial low‐frequency noise (LFN) leads to performance degradation and evolves into a noteworthy reliability concern, primarily because of its characteristic where the amplitude of LFN magnifies with device miniaturization.^[^ ^26^ ^]^

The interface between metal and 2D semiconductors represents a prominent source of excess noise in 2D FETs.^[^ ^12^, ^13^, ^14^, ^15^, ^17^, ^21^, ^23^ ^]^ While the theoretical anticipation suggests that 2D semiconductors should lack covalent‐bond‐based surface interactions, real‐world scenarios demonstrate that charge transport in the majority of 2D FETs is predominantly controlled by a Schottky barrier height (SBH) and Fermi level pinning (FLP) at the metals‐2D semiconductor contact.^[^ ^27^, ^28^ ^]^ Directly depositing metal onto a 2D semiconductor results in the accumulation of adsorbed water or hydrocarbon layers on the surface. These contaminants introduce interface states, resulting in FLP. Furthermore, when in direct contact with bulk metal like Au, the contact region experiences substantial lattice strain, leading to damage, and defect generation.^[^ ^27^ ^]^ All these factors culminate in the generation of excess noise in 2D FETs. Addressing the contact resistance issue in 2D FETs and mitigating the excess noise generated from this contact region is of paramount importance. This is particularly crucial because the carrier transport in short‐channel 2D FETs is almost ballistic, and the majority of power dissipation occurs at the contacts. A promising solution to tackle this issue involves forming a clean van der Waals junction.^[^ ^29^, ^30^ ^]^ Van der Waals junction through metal transfer onto 2D semiconductors can establish a clean interface without causing damage to the underlying 2D material. However, achieving a clean van der Waals junction still remains challenging due to the interfacial interaction between metal atoms with high thermal or impinging energy and the 2D semiconductor, which can disrupt the van der Waals gap. Hence, it is imperative to discover methods to form clean vdW contacts, and their corresponding effects on LFN characteristics need to be systematically demonstrated in 2D FETs.

Furthermore, defects like vacancies, grain boundaries, and agglomerated line vacancies exert significant compromise on the reliability and degradation of the LFN in 2D FETs.^[^ ^31^, ^32^, ^33^ ^]^ In 2D semiconductors with sulfide elements, such as molybdenum disulfide (MoS_2_), sulfur vacancies pose a pronounced reliability issue.^[^ ^31^ ^]^ Common initiating factors, including water oxygen stemming from the manufacturing processes and in the operational environment of MoS_2_ FETs, can readily trigger the progression of vacancy defects in MoS_2_. This phenomenon stands as a pivotal hazard to reliability. Understanding the developmental trajectory of defects stemming from these initiators takes precedence, as it is crucial for comprehending their impact on FET reliability and LFN characteristics. However, previous research on LFN in 2D FETs has primarily focused on analyzing the impact of defects at the interface between the channel material and the gate oxide or within the bulk of the gate oxide.^[^ ^11^, ^12^, ^13^, ^14^, ^15^, ^16^, ^17^, ^18^, ^19^, ^20^, ^21^, ^22^, ^23^, ^24^, ^25^ ^]^ This analysis falls short of providing a comprehensive understanding of the LFN characteristics of 2D FETs. In 2D FET, the above‐mentioned vacancies induce subgap density of states (DOS), and they generate excess LFN.^[^ ^34^ ^]^ The influence of vacancy‐induced DOS on the LFN characteristics of 2D FETs should be extensively investigated, along with exploring methods to mitigate this excess noise generated by vacancies. It is important to note that this investigation is of significant importance in chemical vapor deposition (CVD) 2D materials. Even though mechanical exfoliation provides a better material quality than CVD, such as a reduced number of sulfur vacancies, exfoliation has significant drawbacks in terms of large‐scale fabrication. For example, the mechanical exfoliation methods used to produce exfoliated MoS_2_ typically result in small, non‐uniform flakes.^[^ ^35^ ^]^ This lack of uniformity and the microscopic scale of the flakes make this method less suitable for large‐scale industrial applications where consistent and uniform material properties are necessary. In contrast, CVD can produce large‐area MoS_2_ layers with greater uniformity. This makes CVD MoS_2_ more suitable for scalable production and applications in industries where large, consistent, and high‐quality materials are required. In this regard, there have been extensive research efforts to improve the quality of CVD MoS_2_, encompassing the analysis of sulfur vacancy‐induced instability and methods to reduce it.^[^ ^36^, ^37^, ^38^ ^]^ However, there is a lack of studies on the investigation of sulfur vacancy on the excess noise of CVD MoS_2_ FETs.

In this study, we investigate the effects of van der Waals junction and sulfur vacancy on the LFN characteristics of monolayer MoS_2_ FETs grown through CVD. We aim to understand the mechanisms underlying the generation of excess noise and explore methods to reduce it, ultimately achieving an ideal LFN behavior in the 2D FETs. To accomplish this, we systematically examine the impact of contact resistance and the DOS on the LFN characteristics. Specifically, we compare the noise performance of MoS_2_ FETs with Au and CuS electrodes and MoS_2_ FETs with CuS electrodes with and without a thermal annealing process. Our findings reveal that the contact noise in the high *I*_D_ region can be significantly reduced by using CuS instead of Au electrodes. Furthermore, through thermal annealing, the presence of sulfur vacancy‐related DOS in the MoS_2_ material is substantially diminished, leading to a reduction in excess noise in the low *I*_D_ region. By optimizing both the metal‐2D material contact and the DOS of the MoS_2_ FETs, we demonstrate that the 1/*f* noise of the device aligns with the pure carrier number fluctuation (CNF) model. This means no excess noise is present in five orders of magnitude in the *I*_D_ and four in the frequency domain. Through our comprehensive investigation and optimization efforts, we achieve the ideal LFN behavior in monolayer MoS_2_ FETs. Our findings highlight the significance of controlling the metal‐2D material contact and addressing the sulfur vacancy‐related DOS to minimize excess noise, paving the way for improved performance and reliability of 2D electronic devices.

## 2 Results and Discussion

**Figure 1:**
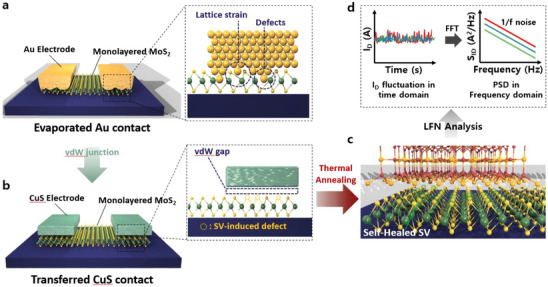
Schematic illustration of the MoS_2_ FET with a) Au, b) CuS electrodes without thermal annealing, and c) CuS electrodes without thermal annealing. d) Schematic illustration of PSD measurement system for these devices. All these devices undergo LFN investigation by transforming fluctuations in drain current (*I*_D_) in the time domain into the frequency domain, manifested as PSD.

This study delves into the LFN characteristics in MoS_2_ FETs by fabricating three distinct device types. The primary aim is twofold: first, to understand the impact of non‐ideal metal‐2D semiconductor contact on LFN characteristics, which is scrutinized by utilizing two different metals for the source and drain electrodes (CuS and Au). Second, it seeks to demonstrate the effects of sulfur vacancy‐induced subgap DOS on MoS_2_ FETs, which is achieved by examining the LFN of the devices with CuS electrodes and subjecting them to an additional annealing process. **Figure** **1**a,b schematically illustrate the MoS_2_ FETs with evaporated Au and transferred CuS electrodes, respectively. Notably, the device with CuS contact showcases the presence of a van der Waals junction, while the device with Au exhibits the effects of interface damage by non‐ideal contact. Figure 1c shows the MoS_2_ FET with thermal annealing. The effects of the annealing are investigated from the perspective of sulfur vacancy healing. All these devices undergo LFN investigation by transforming fluctuations in drain current (*I*_D_) in the time domain into the frequency domain, manifested as power spectral density (PSD), as shown in Figure 1d.

The fabrication process of the devices is as follows: The Au/MoS_2_ FETs were fabricated by depositing the Au through the thermal evaporator onto the CVD growth MoS_2_, which had been transferred onto the 20 nm HfO_2_ substrate. On the other hand, the CuS/MoS_2_ FETs were fabricated by transferring the pre‐patterned CuS electrode onto the transferred MoS_2_. Notably, the self‐healing effect by the CuS electrode on the MoS_2_ was achieved through a thermal annealing process of the CuS/MoS_2_ transistor at 150 °C under the vacuum condition for 1 h.^[^ ^39^ ^]^ A detailed explanation of the fabrication is given in the Experimental Section. To verify the monolayer thickness and high crystal quality of the CVD growth MoS_2_, we first conducted Raman and photoluminescence (PL) spectroscopy. Figure S1a (Supporting Information) shows the Raman spectrum of the CVD growth MoS_2_. The difference between the distinctive Raman peak of MoS_2_ crystal (E^1^_2g_ and A_1g_ Raman mode) was ≈19 cm^−1^, which confirms the monolayer thickness of MoS_2_.^[^ ^40^ ^]^ In addition, the PL spectra reveals a sharp peak at 1.84 eV (Figure S1b, Supporting Information), which means that our CVD growth MoS_2_ has monolayer thickness, direct bandgap nature, and excellent crystal quality. In addition, the thickness of MoS_2_ was confirmed to be ≈0.7 nm through the height profile of the atomic force microscope (AFM) mapping image, as shown in Figure S2 (Supporting Information).^[^ ^41^ ^]^ To confirm the structure of the CuS electrode, which we synthesized by using the atmospheric sulfurization method, we performed the XRD analysis (Figure S3, Supporting Information). The XRD spectra distinctly display the covellite CuS structure (JCPDS Card No. 06–0464) among the variety of binary phases of copper sulfides such as chalcocite, djurlite, digenite, anilite, or covellite phases.^[^ ^42^, ^43^, ^44^, ^45^ ^]^ Figure S4 (Supporting Information) shows the optical images of the fabricated FETs. Note that the length of the devices is identical to 5.1µm. The widths of the devices with Au and CuS electrodes are 27.98 and 17.32 µm, respectively. Information about the break‐down electric field, capacitance, and dielectric constant are shown in Figure S5 (Supporting Information).

The goal of this study is to realize the ideal LFN characteristics of MoS_2_ FETs. In this study, ideal LFN signifies a state where the noise source of the device is solely determined by a single factor. This implies that the conduction mechanism should be dictated by a single factor, resulting in a predictable noise source. When the LFN characteristics of the device follow the CNF model in entire operating regions, it demonstrates that the conduction of the FET is determined by the carrier flow occurring at the interface between the gate dielectric and channel, verifying good gate controllability. Non‐idealities, such as the substantial influence of factors like contact resistance and sulfur vacancy‐induced defects, pose challenges to achieving this ideal LFN behavior. In instances where these non‐idealities play a prominent role, the electrical performance of the device is compromised, leading to the generation of excess noise. An analogous example is the correlated mobility fluctuation model (CMF), which explains noise behavior in scenarios where trapped charge induces Coulombic scattering, acting as an additional noise source. In the context of 2D FETs, a major concern is the excess noise induced by metal‐semiconductor contact. Ideally, conduction should remain unaffected by contact resistance. However, a shift in the noise source from the CNF to contact signifies a change in the conduction mechanism, with the contact dominating the carrier transport process. This shift to contact resistance as the primary noise source indicates a non‐ideal conduction process in 2D FETs. A parallel logic can be applied to explain the influence of subgap DOS on LFN. When a significant number of sulfur vacancies are present, trapping from/to DOS generates excess noise and hampers carrier mobility, leading to a non‐ideal conduction mechanism and LFN. In this study, we optimize the metal electrode and employ a thermal annealing process. These approaches result in fabricating a device where conduction is singularly determined by the carrier flow through the interface between MoS_2_ and HfO_2_ in both, ultimately achieving ideal LFN characteristics.

**Figure 2:**
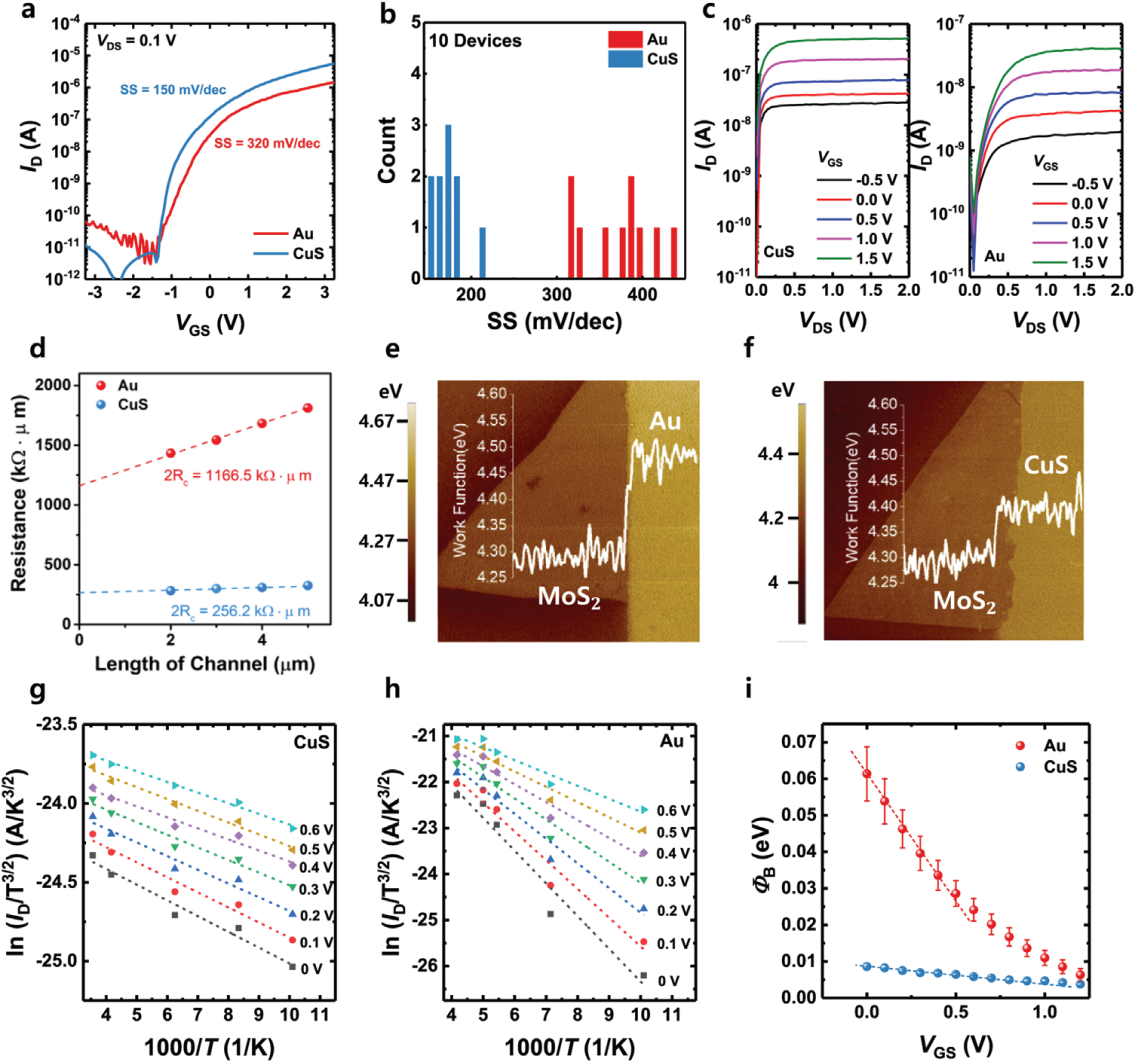
a) *I*_D_–*V*_GS_ of the MoS_2_ FETs with Au and CuS electrodes with a *V*_DS_ of 0.1 V. b) SS values of the ten independent devices with different electrodes. c) Output curve of the FET with CuS and Au electrodes. The gate voltage (*V*_GS_) was in the range from 1.5 to −0.5 V with a 0.5 V step. d) Total resistance as a function of channel length. From the y‐intercept of the linear fit, the 2R_c_ of the FETs is extracted. KPFM images of the e) MoS_2_/Au junction and f) MoS_2_/CuS junction, respectively. The significant band offset between the MoS_2_ and Au electrode junction, forming a SBH. Arrhenius plot for MoS_2_ FET with g) CuS and h) Au electrodes. i) *Φ*_B_ value versus *V*_GS_ of MoS_2_ FETs with Au and CuS electrodes, respectively.

First, we compare the material, electrical, and LFN characteristics of the MoS_2_ FETs with CuS and Au electrodes. **Figure** **2**a shows the transfer characteristics (*I*_D_–*V*_GS_) of the MoS_2_ FETs with Au and CuS electrodes with a fixed drain‐to‐source bias voltage (*V*_DS_) of 0.1 V. It is clearly observed that the device with CuS electrode exhibits superior electrical characteristics, including higher on‐current (*I*_on_), enhanced transconductance (*g*_m_), and improved subthreshold swing (SS). Furthermore, hysteresis is reduced in the device with a CuS electrode. Figure 2b shows the SS values of the ten independent devices with different electrodes. Note that other electrical characteristics, including threshold voltage, field‐effect mobility, and interface trap density (*N*_it_) determined by subthreshold are shown in Table S1 (Supporting Information). Figure 2c shows the output characteristics (*I*_D_–*V*_DS_) of the FET with CuS and Au electrodes with an increase in *V*_GS_ from −0.5 to 1.5 V in increments of 0.5 V. Notably, the device with CuS electrode exhibits stable saturation behavior, further highlighting its superior performance. To demonstrate such differences in electrical characteristics, transfer length method (TLM) measurement is conducted using channel lengths of 2, 3, 4, and 5 µm.^[^ ^46^ ^]^ In Figure 2d, the total resistance is plotted as a function of channel length, and the y‐intercept of the linear fit is defined as the contact resistance (2R_c_) of the FETs. The 2R_c_ of the FETs with Au electrode (1166.5 kΩ·µm) was found to be approximately five times higher than the FETs with CuS electrode (256.2 kΩ·µm). Here, it is important to find the reason for such a decrease in contact resistance. Figure 2e,f shows the work function mapping at the MoS_2_ channel and electrode interface. The Kelvin probe force microscopy (KPFM) images clearly show the significant band offset between the MoS_2_ and Au electrode junction, forming a high barrier height (Figure 2e). However, a decrease in the difference in the work function is observed in the MoS_2_ and CuS electrode interface (Figure 2f). These results demonstrate that the work function offset can lead to a decreased contact barrier in the device with the CuS electrode, resulting in improved electrical properties. The reduced contact barrier of the FETs with the CuS electrode is also elucidated by extracting the Schottky barrier height (SBH) from the thermionic emission current. The thermionic emission current density is described by the 2D thermionic emission equation:^[^ ^47^ ^]^

(1) $$I_{D}=\;A{A_{2D}}^{*}T^{3/2}\exp\left[-\frac{q}{kT}\left(\Phi_{B}-\frac{V_{\mathrm{DS}}}{\eta }\right)\right]$$

where *A* is the contact area, *A*_2D_^*^ is the 2D equivalent Richardson constant, *q* is the electron charge, *Φ*_B_ is the SBH, *k* is the Boltzmann constant, and *η* is the ideality factor. The SBH can be extracted from the slope of an Arrhenius plot of ln(*I*_D_/*T* ^3/2^) versus 1000/*T* for each FET (Figure 2g,h). The SBH is determined at a gate voltage corresponding to the flat band condition of the MoS_2_ FETs. The extracted *Φ*_B_ is plotted as a function of applied *V*_GS_ in Figure 2i. We define SBH as a point where *Φ*_B_ value starts to depart from the linear dependence of the gate voltage. As a result, the SBHs for the FETs with CuS electrode and Au electrode are found to be ≈4.8 and ≈30 meV, respectively, which is consistent with our various analyses on contact resistance.

**Figure 3:**
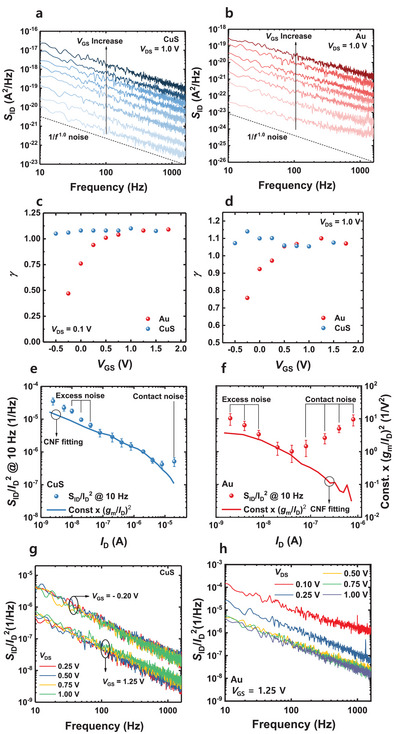
*S*_ID_ versus frequency for the MoS_2_ FETs with a) CuS and b) Au electrodes, respectively. The *V*_DS_ is set at 1.0 V during the measurement, and the *V*_GS_ is increased to investigate the LFN behavior in different operating regions. The slope of the PSD (*γ*) for FETs with Au and CuS electrodes was measured at *V*_DS_ of c) 0.1 and d) 1.0 V, respectively. *S*_ID_/*I*_D_^2^ values sampled at 10 Hz and (*g*_m_/*I*_D_)^2^ values versus *I*_D_ of the MoS_2_ FETs with e) CuS and f) Au electrodes, respectively. g) *S*_ID_/*I*_D_^2^ versus frequency of the MoS_2_ FET with CuS electrodes measured at different *V*_GS_ (−0.20 and 1.25 V) and *V*_DS_ (0.25, 0.50, 0.75, and 1.0 V) values. h) *S*_ID_/*I*_D_^2^ versus frequency of the MoS_2_ FET with Au electrodes measured at different *V*_DS_ values (0.1, 0.25, 0.50, 0.75, and 1.0 V).

Next, we proceed with LFN analysis of the MoS_2_ FETs. To ensure the accuracy of the PSD measurement in 2D FETs, it is important to account for any potential current drift that may affect the validity of the PSD measurement. Therefore, we investigate the effects of current drift during the PSD measurement process. Figure S6a,b (Supporting Information) shows the DC‐measured *I*_D_–*V*_GS_ characteristic curves and *I*_D_–*V*_GS_ values during the LFN measurement of the devices with CuS and Au electrodes, respectively, demonstrating nearly identical behavior. This indicates that the current drift has a negligible impact on the LFN measurement. Additionally, Figure S6c (Supporting Information) shows the transient *I*_D_ characteristics during the PSD measurement, revealing negligible current drift. These findings suggest that the effects of current drift on the LFN measurement can be safely disregarded. **Figure** **3**a,b shows the *I*_D_ PSD (*S*_ID_) for the FETs with CuS and Au electrodes, respectively. The *V*_DS_ is set at 1.0 V during the measurement, and the *V*_GS_ is increased to investigate the LFN behavior in different operating regions. Note that the PSD for *V*_DS_ at 0.1 V is shown in Figure S7a,b (Supporting Information). Figure S8a (Supporting Information) shows the ten times of repeated measurement results of the PSD. The bold black line represents the average value. The PSDs exhibit reproducible results, demonstrating the reliability of the measurement system. Figure S8b (Supporting Information) shows the statistics of the *S*_ID_ value sampled at 10, 10^2^, and 10^3^ Hz. With an increase in *V*_GS_, the *S*_ID_ increases in both devices, indicating that the magnitude of the current fluctuation increases with a higher *I*_D_. In both devices, the PSD exhibits the 1/*f* ^γ^ noise behavior (*γ* = −∂ln/∂*f*). Figure 3c,d shows the *γ* values versus *V*_GS_ for the FETs with Au and CuS measured at *V*_DS_ of 0.1 and 1.0 V, respectively. The FETs with CuS exhibit the constant *γ* value of 1 across all operating regions, indicating a uniform distribution of trap density. In contrast, the FETs with Au exhibit an operating region‐dependent *γ* behavior, suggesting a non‐uniform distribution of the trap density across the energy and spatial regions. The difference in the *γ* value becomes more pronounced when a lower *V*_DS_ is applied to the devices. The FET with Au electrode shows a significantly smaller γ value at the low *I*_D_ region. This result further emphasizes the impact of contact resistance in the FET with Au electrode, particularly for the low *V*_DS_ conditions.

To explore the underlying source of the 1/*f* noise behavior in the FETs, the *S*_ID_ is normalized to the values of *I*_D_ at each measurement point. The resulting *I*_D_ normalized PSD (*S*_ID_/*I*_D_^2^) values are then compared to (*g*_m_/*I*_D_)^2^ as a function of *I*_D_, as depicted in Figure 3e,f. The *V*_DS_, *V*_GS_, and *I*_D_ values for PSD measurements for MoS_2_ FETs with CuS and Au electrodes are shown in Figure S6a,b (Supporting Information). This analysis aims to determine whether the 1/*f* noise in the FETs is governed by the CNF model. In FETs, a significant number of carriers can be captured or emitted by defects within the gate oxide, leading to fluctuations in the flat‐band voltage (*V*_fb_) and, subsequently, fluctuations in *I*_D_.^[^ ^48^, ^49^, ^50^, ^51^ ^]^ The CNF model is expressed as

(2) $$\frac{S_{\mathrm{ID}}}{{I_{D}}^{2}}={\left(\frac{g_{m}}{I_{D}}\right)}^{2}\;S_{\mathrm{Vfb}}$$

where *S*_Vfb_ is the *V*_fb_ fluctuation. The *S*_Vfb_ is expressed as

(3) $$S_{\mathrm{Vfb}}=\frac{q^{2}k_{B}TN_{T}\lambda }{WL{C_{\mathrm{ox}}}^{2}f}\;\;$$

where *q* is the electron charge, *k*_B_ is the Boltzmann constant, *T* is the temperature, *N*_T_ is the volume trap density, λ is the tunneling attenuation coefficient, *WL* is the effective area of the gate, and *C*_ox_ is the gate oxide capacitance per unit area, and *f* is the frequency. Both devices exhibit the deviations of S_ID_/*I*_D_^2^ from the (*g*_m_/*I*_D_)^2^ in both the low and high *I*_D_ regions, indicating the presence of excess noise in the devices. Note that the analysis of excess noise in the low *I*_D_ region will be addressed later, specifically in the analysis of LFN characteristics of MoS_2_ FETs with thermal annealing. For now, we focus on the excess noise in the high *I*_D_ region. It is widely recognized that the presence of excess noise in the high *I*_D_ region is primarily attributed to contact resistance.^[^ ^12^, ^13^, ^14^, ^15^, ^17^, ^21^, ^23^ ^]^ The impact of contact resistance becomes evident with an increase in the *V*_GS_ (increase in *I*_D_), and the 1/*f* noise generated by the barrier height fluctuation (BHF) at the metal‐semiconductor contact plays a dominant role in determining the overall LFN characteristics of the FETs. In particular, various types of defects, including sulfur vacancies, at the metal/2D materials contact increases the BHF.

To verify that the excess noise in the high *I*_D_ regions originates from the metal/2D semiconductor contact, the PSD is measured at various *V*_DS_ values while changing the *V*_GS_. Figure S9 (Supporting Information) shows the log–log plot of the S_ID_/*I*_D_^2^ values sampled at 10 Hz versus *V*_DS_ of FETs with CuS and Au electrodes. Note that two different *V*_GS_ values are used: *V*_GS_ = 0.25 V to represent the low *I*_D_ region and *V*_GS_ = 1.25 V to represent the high *I*_D_ region. In the case of FET with CuS, the S_ID_/*I*_D_^2^ exhibits little dependence on *V*_DS_ in both *V*_GS_ values. Figure 3g shows the S_ID_/*I*_D_^2^ as a function of the frequency of the FET with CuS electrode with *V*_GS_ = − 0.20 and 1.25 V as a parameter of *V*_DS_. In both *V*_GS_ values, the S_ID_/*I*_D_^2^ does not exhibit *V*_DS_ dependence, demonstrating the negligible effect of contact resistance. In contrast, the FET with Au electrode shows different *V*_DS_ dependence depending on the *V*_GS_ values (Figure 3h; Figure S9b, Supporting Information). Whereas the device exhibits no dependence on *V*_DS_ in the low *V*_GS_ value (Figure S9b, Supporting Information), there is a significant increase in the noise at the low *V*_DS_ region in the high *V*_GS_ value (Figure 3h). This is because the impact of excess noise is more severe as the number of carriers is increased with an increase in *V*_GS,_ and thus, the CNF at the channel is decreased. Accordingly, BHF at the 2D material/metal electrode interface becomes more prominent. In particular, when the *V*_DS_ is low, the effective barrier height is larger, and thus the BHF is large. This is why significant excess noise is observed in the low *V*_DS_ and high *V*_GS_ regions of FETs with Au electrodes. These findings are consistent with the material analysis performed on the MoS_2_ FETs as illustrated in Figure 2e,f. The outcomes clearly demonstrate the substantial reduction in the influence of contact resistance in the MoS_2_ with CuS electrodes. This reduction is attributed to the van der Waals junction, which diminishes the impact of interface damage on the metal‐2D semiconductor junction, consequently leading to a decrease in the presence of defect states.

Additionally, we investigate the electrical and LFN characteristics of the MoS_2_ FET with transferred Au electrode to demonstrate the effects of electrode material. Figure S10a (Supporting Information) shows the optical images of the fabricated MoS_2_ FET with transferred Au electrode. Figure S10b (Supporting Information) shows the *I*_D_–*V*_GS_ of the MoS_2_ FET with transferred Au electrode and other FETs. A similar SS value is observed between the MoS_2_ FETs with evaporated and transferred electrodes. However, the *I*_on_ of the device with transferred Au electrode is larger than that with evaporated Au. This result demonstrates that the contact resistance is decreased by the adoption of a transfer Au electrode. Figure S10c (Supporting Information) shows the *S*_ID_/*I*_D_^2^ and (*g*_m_/*I*_D_)^2^ versus *I*_D_ of the transfer Au MoS_2_ FET. The effects of contact resistance on the excess noise in the high *I*_D_ region are decreased by the transfer process, demonstrating that enhancing the quality of metal‐semiconductor junction through van der Waals contact mode is crucial for achieving ideal LFN characteristics. However, even though the contact resistance is improved by the transfer process, the effects of contact noise in the high *I*_D_ region persist. Moreover, the electrical and LFN characteristics of the MoS_2_ FET with transferred Au electrode are poorer than those of MoS_2_ FET with transferred CuS electrode. These findings indicate that not only the contact mode, whether it is a van der Waals junction or not, but also the electrode material plays a significant role in determining the ideal LFN characteristics. The choice of metal material contributes significantly by determining the work function of the metal. Opting for CuS over Au is advantageous, considering the bandgap offset, which forms a lower barrier height at the metal‐semiconductor junction (Figure 2e,f). Therefore, it can be concluded proper choice of both contact mode and electrode material is crucial.

**Figure 4:**
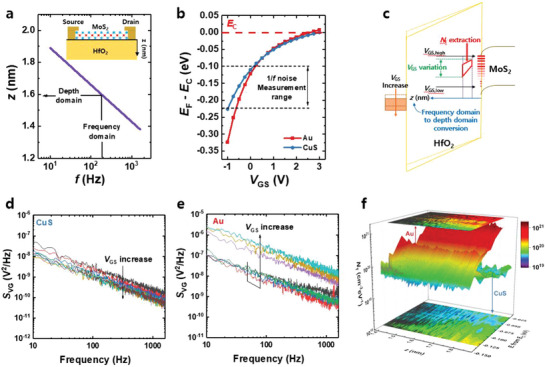
a) *z* versus *f* of the MoS_2_ FETs. b) Precise energy distribution, specifically *E*_F_‐*E*_C_, as a function of *V*_GS_ of the MoS_2_ FETs with Au and CuS electrodes. c) Schematics of the energy diagram of the MoS_2_ FET considering the non‐uniformity of the defects. *S*_Vfb_ as a function of frequency for various *V*_GS_ values of the FETs with d) CuS and e) Au electrodes, respectively. f) 3D plot for the spatial and energetic distribution of *N*_T_ extracted from PSD for FETs with CuS and Au electrodes.

Based on the obtained result, we profile the defects within the MoS_2_ FETs. Previous studies on LFN characterization of 2D FETs that employed the CNF model commonly assume a uniform distribution of defects in both spatial and energetic aspects. However, in the case of 2D FETs, the LFN behavior is significantly influenced by the non‐uniform distribution of defects. Specifically, the defects within the gate oxide exhibit a spatial distribution that is non‐uniform, and this characteristic is reflected in the slope of PSD (*γ*). The frequency components of the PSD are dependent on the depth of the defects, which governs the trapping and detrapping processes responsible for 1/*f* noise. When the trap is located farther from the interface between the gate oxide and the channel material, the trapping, and detrapping constants are larger, resulting in a steeper slope of the 1/*f* noise. Conversely, when the defects are more concentrated near the interface, the slope becomes smaller than one. The frequency domain in PSD can be thus transited to the depth domain of the gate oxide, and this relationship is described as^[^ ^52^ ^]^

(4) $$z\;=\;\lambda \ln\left(\frac{1}{2\pi f\tau_{0}}\right)\;$$

where τ_0_ is the time for tunneling into a trap state at the interface (*z* = 0). **Figure** **4**a shows the relationship between the distance from the interface to the bulk direction and the frequency range.

Another important factor to consider is the non‐uniform distribution of defects in the energy domain. The CNF model typically assumes a uniform distribution of defects across the energy levels. However, this assumption does not hold for materials such as amorphous oxide semiconductors or 2D materials, where the presence of DOS is significant in the subgap. The energy levels of the defects that contribute to the 1/*f* noise in the device vary depending on the magnitude of *V*_GS_. As *V*_GS_ increases, the energy level associated with the conduction band energy (*E*_C_) approaches the Fermi level (*E*_F_), and it becomes the dominant factor governing the 1/*f* noise behavior. To establish the relationship between the relative energy of the *E*_C_ and the *V*_GS_, it is crucial to accurately map *E*_C_ to *V*_GS_ by matching the corresponding gate voltage. This relationship can be derived using the following equation:^[^ ^53^ ^]^

(5) $$I_{D}\left(V_{\mathrm{GS}}\right)\;=\;AqN_{C}\mu_{\mathrm{eff}}E\exp\left(\frac{E_{F}\left(V_{\mathrm{GS}}\right)-E_{C}}{kT}\right)$$

where *A* represents the conducting channel area, *N*_C_ is the density of states in the conduction band, *µ*_eff_ is the effective mobility, *E* is the electrical field, and *q* is the elementary charge. Figure 4b shows the precise energy distribution, specifically *E*_F_‐*E*_C_, as a function of *V*_GS_. Figure 4c shows the schematics of the energy diagram of the MoS_2_ FET considering the non‐uniformity of the defects.

Based on this analysis, the profile of defects within the devices can be determined. To evaluate the *N*_T_, the *S*_Vfb_ is calculated. Note that the *S*_Vfb_ value extracted at the operating region where the 1/*f* noise is not generated by the CNF can be numerically inaccurate. However, this value can be used for comparative analysis, providing insight into the magnitude of the increased surplus *S*_Vfb_ when excess noise contributes to the CNF. Figure 4d,e illustrates the *S*_Vfb_ as a function of frequency for various *V*_GS_ values of the FETs with CuS and Au electrodes, respectively. For the FET with CuS electrode, the *S*_Vfb_ shows a consistent behavior across both frequency and *V*_GS_, with a slight increase in the low *V*_GS_ region. This indicates a relatively uniform distribution of defects in both energy and space domains. In contrast, the FET with Au electrode exhibits a significant increase in *S*_Vfb_ in the high *V*_GS_ region and a distinct difference in the slope of the curve. This points to a non‐uniform distribution of defects within the device. Figure 4f shows the 3D plot for the spatial and energetic distribution of *N*_T_ extracted from PSD for FETs with CuS and Au electrodes. Figure S11a,b (Supporting Information) shows the *N*_T_ versus *z* and *E* of the MoS_2_ FETs with Au and CuS electrodes, respectively. Note that the excess noise from the BHF at the contact is also included in this *N*_T_ extraction. The *N*_T_ values are comparable in the case where the CNF is the main noise source (*E*_F_‐*E*_C_ = −0.08 eV; 5.4  ×  10^19^ and 4.30  ×  10^19^ cm^−3^ eV^−1^ for FETs with Au and CuS electrodes), demonstrating a similar MoS_2_/HfO_2_ interface quality. However, the FET with Au electrode exhibits a much larger *N*_T_ than the FET with CuS electrode when the excess noise exists. This difference in *N*_T_ is particularly pronounced near the *E*_C_, indicating the presence of defects at the MoS_2_/Au contact. Through the analysis conducted, it can be concluded that the use of CuS electrodes significantly reduces the *N*_T_ associated with contact resistance. This reduction in *N*_T_ contributes to improved device performance, highlighting the importance of realizing the van der Waals junction for achieving ideal LFN characteristics.

**Figure 5:**
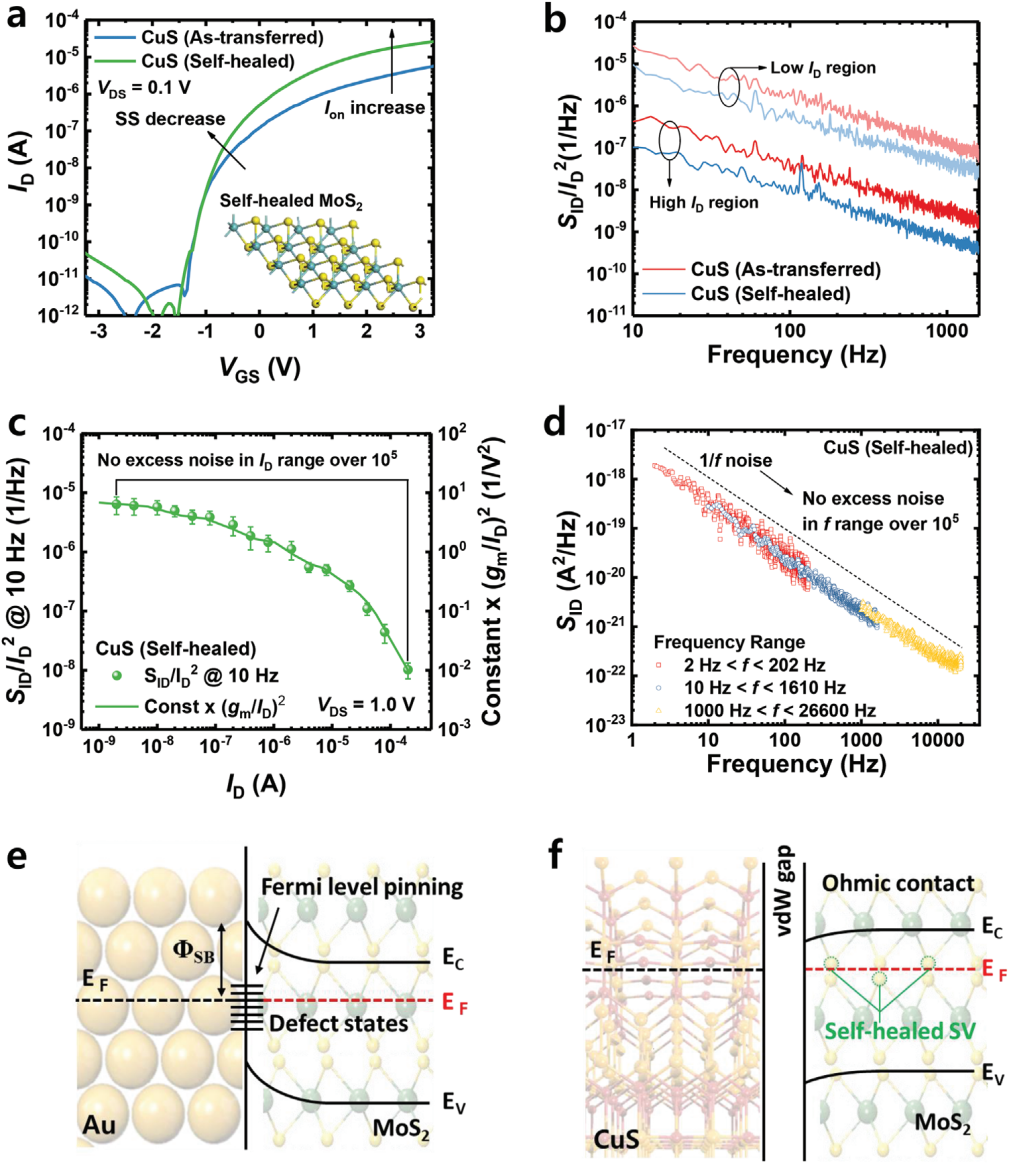
a) *I*_D_–*V*_GS_ curves of the MoS_2_ FETs with CuS electrode, comparing the devices with and without the annealing process. b) *S*_ID_/*I*_D_^2^ versus frequency of the CuS electrode FET with and without thermal annealing. c) *S*_ID_/*I*_D_^2^ values sampled at 10 Hz and (*g*_m_/*I*_D_)^2^ values versus *I*_D_ of the MoS_2_ FETs with thermal annealing. Remarkably, throughout this entire range, the behavior of *S*_ID_/*I*_D_^2^ follows that of (*g*_m_/*I*_D_)^2^, indicating the absence of any excess noise. d) PSD within different frequency ranges: 2 Hz < *f* < 202 Hz, 10 Hz < *f* < 1610 Hz, and 1000 Hz < *f* < 2.66 × 10^4^ Hz. e) and f). Schematic representation of the proposed strategy aimed at diminishing excess noise and attaining ideal 1/*f* noise behavior in MoS_2_ FETs.

Now, we move on to the LFN characteristics of the device with the annealing process. **Figure** **5**a shows the *I*_D_–*V*_GS_ curves of the MoS_2_ FETs with CuS electrode, comparing the devices with and without the annealing process. Interestingly, the decrease in SS and increase in *I*_on_ are observed after the annealing. Note that the device with CuS electrode with thermal annealing exhibits hysteresis‐free behavior. Improvement in electrical properties with thermal annealing has been reported.^[^ ^39^ ^]^ However, the corresponding change in the LFN characteristics has not been demonstrated. Figure 5b shows the *S*_ID_/*I*_D_^2^ versus frequency of the CuS electrode FET with and without thermal annealing. The decrease of the 1/*f* noise in the high *I*_D_ region is observed, demonstrating the improvement in the metal/2D material interface. The improvement in device performance can be attributed to the self‐healing mechanism of sulfur vacancies. Excess sulfur adatoms from the CuS electrodes diffuse through the MoS_2_ channel and interact with the defect sites, such as sulfur vacancies. This interaction leads to the spontaneous healing of the vacancies through the thermodynamically favorable adsorption and binding of the sulfur adatoms. As a result, the presence of sulfur vacancies is reduced, leading to an enhanced MoS_2_/CuS electrode interface.

Furthermore, a reduction in 1/*f* noise within the low *I*_D_ range is observed. To elucidate the cause of this reduction, the *S*_ID_/*I*_D_^2^ and (*g*_m_/*I*_D_)^2^ are plotted against *I*_D_, covering a range of 2 nA to 200 µA, spanning over 10^5^ *I*_D_ values. Remarkably, throughout this entire range, the behavior of *S*_ID_/*I*_D_^2^ follows that of (*g*_m_/*I*_D_)^2^, indicating the absence of any excess noise (Figure 5c). Additionally, we measure the PSD of the CuS electrode FETs with thermal annealing in the frequency range of 2 to 2.66 × 10^4^ Hz, as depicted in Figure 5d. Even in the frequency domain, the PSD exhibits consistent 1/*f* ^γ^ noise (*γ* = 1) without any presence of a Lorentzian bulge or a change in slope. Note that these results are obtained by performing three separate measurements of the PSD within different frequency ranges: 2 Hz < *f* < 202 Hz, 10 Hz < *f* < 1610 Hz, and 1000 Hz < *f* < 2.66 × 10^4^ Hz, all using the same gain for the low noise current preamplifier. Due to the negligible current drift during the PSD measurement, the PSD curves from the three frequency ranges perfectly overlap. This ideal 1/*f* noise behavior is attributed to the self‐healing during thermal annealing. As aforementioned, during thermal annealing, the adatoms from the CuS electrodes diffuse through the MoS_2_ channel and fill the sulfur vacancies. Accordingly, the DOS regarding the sulfur vacancy is significantly reduced by thermal annealing. To confirm the CuS‐induced self‐healing effect on the MoS_2_ through the mild annealing process, we employed XPS analysis as shown in Figure S12 (Supporting Information). The XPS spectra present the peaks corresponding to the Mo 3d orbital (Mo+4 3d5/2 and Mo+4 3d3/2) of the pristine MoS_2_ and the self‐healed MoS_2_. After the self‐healing process, the deconvolution peak intensity of i‐MoS_2_ (Intrinsic MoS_2_) is increased, whereas d‐MoS_2_ (defective MoS_2_) decreases, which confirms the self‐healing effect on the MoS_2_ crystal. It is also found evidence of the self‐healing effect within the XPS spectra of the S 2p peak ‐which is a consistent result with our previous report (Figure S13, Supporting Information).^[^ ^39^ ^]^ It should be noted that sulfur vacancy defects, which are the most prevalent defects, are known to create defect states within the bandgap close to the conduction band. The creation of defect states in the subgap is also confirmed using density functional theory (DFT) calculations. Figure S14 (Supporting Information) shows the band structure of the MoS_2_ crystal with different numbers of sulfur vacancies. The band structure is calculated by using first‐principle calculations (see Experimental Section for details). For the perfect MoS_2_ crystal, no states in the bandgap were observed. However, when single or double sulfur vacancy is introduced on the surface of the MoS_2_ crystal, additional energy states emerge within the forbidden region of the band structure.

Based on these findings, it can be inferred that the excess noise observed in the low *I*_D_ region can be attributed to the presence of sulfur vacancy‐induced DOS. Specifically, the double sulfur vacancy primarily generates DOS within the forbidden energy gap, with energy levels formed ≈0.2–0.3 eV away from the *E*_C_ (Figure S14, Supporting Information). As depicted in Figure 4b, this energy range corresponds to the measurement range for 1/*f* noise, particularly in the low *I*_D_ region. When subgap DOS exists in the channel material, the carriers within the channel undergo a cycle of trapping/detrapping processes not only with defects inside the gate oxide but also with the DOS within the channel material itself. This leads to excess noise contributions from the CNF, as the CNF solely accounts for processes associated with defects inside the gate oxide. Consequently, this explains the observed excess noise in MoS_2_ FETs without thermal annealing, which exhibit a significant number of sulfur vacancies. However, with thermal annealing, the sulfur vacancy‐induced DOS is self‐healed, resulting in MoS_2_ FETs that display no excess noise in the low *I*_D_ region. It is expected that sulfur vacancy‐induced subgap DOS is eliminated through sulfur diffusion from the CuS electrode during the self‐healing process. Accordingly, the excess noise in the *I*_D_ region disappears, demonstrating perfectly ideal LFN characteristics.

Figure S15 (Supporting Information) shows the *N*_T_ values of the HfO_2_ in MoS_2_ FETs with and without thermal annealing. Note that the *N*_T_ values are compared specifically in the operating region where the LFN characteristics of both devices adhere to the CNF model, as we previously mentioned. This is because the accurate value of *N*_T_ can only be extracted when the 1/*f* noise is determined by the CNF model. Both devices exhibit a similar value of the *N*_T_, indicating that this similarity is attributed to minimal alterations in the HfO_2_ quality during the thermal annealing process at 150 °C. Thus, it can be concluded that the improvement of the electrical properties and LFN characteristics by the thermal annealing process stems from the improvement of MoS_2_ channel characteristics, specifically the reduction of sulfur vacancy, rather than improvements in the gate dielectric characteristics. Figure 5e,f provides a schematic representation of the proposed strategy aimed at diminishing excess noise and attaining ideal 1/*f* noise behavior in MoS_2_ FETs. This approach involves forming a van der Waals junction utilizing a CuS electrode and utilizing thermal annealing to facilitate the self‐healing of sulfur vacancy‐induced DOS. To the best of our knowledge, the result marks the first instance of showcasing the effects of van der Waals junctions and sulfur vacancy in MoS_2_ FETs on LFN characteristics and methods to reduce them. Furthermore, it is noteworthy that the influence of sulfur vacancies on both contact resistance value and LFN characteristics is identified. We illustrated that noise resulting from contact resistance can be diminished through a thermal annealing process that leverages self‐healing effects. The ideal 1/*f* noise behavior achieved in this study markedly outperforms the outcomes reported for previously investigated MoS_2_ FETs, as demonstrated in **Table** **1**. Furthermore, the optimized device demonstrates either lower or comparable *N*_T_ values when compared with these previous studies.

**Table 1:** Comparison of LFN characteristics of MoS_2_ FETs between previous studies and this work.

	Gate Dielectric	Measured *I*_D_ range	Measured *f* range	Noise model (Excess noise)	*N*_T_ [cm^−3^eV^−1^]
[11]	SiNx	5 time	3 orders	CNF (Excess noise in frequency domain)	N/A
[12]	HfLaO	2 orders	3 orders	CNF (No excess)	N/A
[13]	Al2O_3_/HfO_2_	‐	3 orders	CNF (No excess)	1.12 × 10^20^
[14]	SiO_2_	2 orders	‐	CNF (Contact noise in high *I*_D_)	N/A
[15]	SiO_2_	5 times	2 orders	CNF (Contact noise in high *I*_D_)	8.78 × 10^20^
[16]	SiO_2_	2 orders	4 orders	HMF (Excess noise in frequency domain)	N/A
[17]	SiO_2_	1.5 orders	‐	HMF (Contact noise in high *I*_D_)	N/A
[18]	SiO_2_	2.5 orders	2 orders	CNF (No excess)	7.47 × 10^19^
[19]	SiO_2_	5 times	3 orders	CNF (No excess)	9.88 × 10^20^
[20]	Al_2_O_3_	3 orders	3 orders	CNF (Excess noise in frequency domain)	7.86 × 10^20^
[21]	hBN	2 orders	2.5 orders	CNF (Excess noise in frequency domain)	N/A
[22]	hBN	1 order	2 orders	CNF (No excess)	1.24 × 10^20^
[23]	SiO_2_	2 orders	4 orders	CNF (Contact noise in high *I*_D_)	N/A
[24]	SiO_2_	5 orders	3 orders	CNF (No excess)	N/A
[25]	hBN	‐	2 orders	CNF (Excess noise in frequency domain)	2.82 × 10^19^
This work	HfO_2_	5 orders	4 orders	CNF (No excess)	2.64 × 10^19^

## 3 Conclusion

In this study, we have investigated the LFN characteristics of MoS_2_ FETs fabricated using various process parameters and proposed optimization methods for achieving ideal LFN characteristics. One crucial improvement involves substituting the Au electrode with a CuS electrode, which effectively reduces the excess noise in the high *I*_D_ region stemming from BHF at the metal/2D material interface. Furthermore, we implemented thermal annealing on the MoS_2_ FETs with CuS electrodes, leading to the self‐healing of sulfur vacancies and the subsequent elimination of excess noise in the low *I*_D_ region. As a result, the optimized FETs exhibited ideal 1/*f* noise behavior across a wide range of *I*_D_ values (spanning over 10^5^ ranges) and frequency ranges (reaching 10^4^ Hz). The results of this study provide a comprehensive analysis of LFN characteristics in 2D material‐based FETs, offering valuable insights into improving the reliability of devices. Based on these findings, a potential solution to a significant impediment hindering the commercial viability of 2D FETs can be proposed.

## 4 Experimental Section

### Synthesis of Monolayer MoS_2_ Crystal

The monolayer MoS_2_ crystals were grown on a 300nm SiO_2_/Si substrate by utilizing thermal chemical vapor deposition (CVD) method.^[^ ^54^, ^55^ ^]^ MoO_3_ powder (Sigma–Aldrich, > 99.0%) as contained in an alumina crucible, which was dissolved in a NH_4_OH (28–30% solution, Sigma–Aldrich), was placed downstream of the furnace while sulfur powder (Sigma–Aldrich, 99.98%) was placed upstream. The furnace was heated at 800 °C with a continuous flow of H_2_/Ar gas. The entire growth process has occurred with an argon‐filled environment at atmospheric pressure.

### Fabrication of CuS Electrode

The CuS electrode was prepared by exposing the pre‐patterned Cu electrode to H_2_S gas, which was evaporated in the ammonium sulfide solution ((NH_4_)_2_S, 24% aqueous solution) at room temperature. Subsequently, the thermal annealing process at 150 °C in 1 h was carried out to achieve a complete covellite CuS structure. The Cu electrode was deposited by using a thermal evaporator on the Photoresist (PR)‐patterned substrate.

### Fabrication of Au/MoS_2_ and CuS/MoS_2_ Transistors

The CVD‐growth MoS_2_ on the SiO_2_ substrate was transferred using Polystyrene (PS, MW ≈192,000, Sigma–Aldrich) film as a transferring assistant onto the 20nm HfO_2_ substrate. The CuS electrode was also transferred onto the MoS_2_ monolayer crystals to fabricate CuS/MoS_2_ transistors. The PS film, which contained MoS_2_ crystals or CuS electrode was separated by penetrating of DI water between the substrate and PS film. The separated PS film was transferred onto the target substrate and dried in the air for a few hours. After the drying process, the PS film was dissolved in toluene. Meanwhile, to deposit the Au electrode onto the MoS_2_ crystals and fabricate Au/MoS_2_ transistors, a thermal evaporator was utilized.

### Characterization of Monolayer MoS_2_ and CuS

To conduct the Raman and PL analysis, Witec confocal Raman spectroscopy with 532 nm laser was used. The AFM measurement was carried out using XE7 AFM setup of Park Systems. An Al‐coated Si cantilever (PPP‐NCHR) was used to obtain AFM mapping image of a monolayer MoS_2_ crystal. The structure of CuS electrode was characterized using X‐ray diffraction analysis of the Rigaku Model SmartLab.

### DFT Calculation for MoS_2_ Crystal

The band structure of the MoS_2_ crystal with S‐vacancy was calculated using the Material Studio. The generalized gradient approximation (GGA) with the functional of Perdew–Burke–Ernzerhof (PBE) was used. A 4 × 4 supercell of MoS_2_ crystal was constructed to include the single S‐vacancy or double S‐vacancy with 30 Å empty space as a vacuum which guarantees negligible van der Waals interaction with other layers. The calculation was performed with the 3 × 3 × 1 k‐point grid in Brillouin zone to optimize the geometry of each MoS_2_ surface structure and energy cutoff of 510 eV.

### LFN Measurement

A semiconductor parameter analyzer (B1500A) was utilized, a low noise current amplifier (SR570), and a signal analyzer (35670A) for measuring PSD.^[^ ^56^, ^57^ ^]^ The measurement process could be outlined as follows: The voltage supplied to gate electrode was controlled by the B1500A. The output drain current was linked to the SR570, which converted current fluctuations into voltage fluctuations. The dynamic signal from the SR570 was then transformed into a power spectral density using the 35670A. Determination of the measurement system's noise floor was crucial. The current amplifier's noise floor was documented at 4  ×  10^−27^A^2^ Hz^−1^ in low noise mode (as per SR570 manufacturer specifications). The PSD of the measurement system in low noise mode aligned with the manufacturer's specifications. This value significantly lies below the device noise, affirming that the measured PSDs in this study remain unaffected by the measurement system's noise floor. A potential distortion in the PSD of devices might arise due to the limited bandwidth.^[^ ^58^, ^59^, ^60^, ^61^, ^62^, ^63^ ^]^ The internal circuitry of the SR570 conserved both signal amplitude and phase. Given that the SR570's rated bandwidths in low noise mode were 2, 20, and 200 kHz for sensitivities of 100 nA, 1 µA, and 10 µA, respectively, spectral distortion was not expected within the restricted frequency range in this study.

## Conflict of Interest

The authors declare no conflict of interest.

## Supporting information

Supporting Information

## Data Availability

The data that support the findings of this study are available from the corresponding author upon reasonable request.
