# Supplementary material for: Toward Ideal Low‐Frequency Noise in Monolayer CVD MoS2 FETs: Influence of van der Waals Junctions and Sulfur Vacancy Management
Source: Adv Sci (Weinh). 2024 May 21;11(28):2307196. doi: 10.1002/advs.202307196 (PMC11267264; doi:10.1002/advs.202307196)
Supplement: Supplementary file 1 — Supporting Information [file ADVS-11-2307196-s001.pdf]

ADVANCED SCIENCE
Open Access

Supporting Information

for *Adv. Sci.*, DOI 10.1002/advs.202307196

Toward Ideal Low-Frequency Noise in Monolayer CVD MoS_2 FETs: Influence of van der Waals Junctions and Sulfur Vacancy Management

Wonjun Shin, Junsung Byeon, Ryun-Han Koo, Jungmoon Lim, Jung Hyeon Kang, A-Rang Jang, Jong-Ho Lee, Jae-Joon Kim, SeungNam Cha*, Sangyeon Pak* and Sung-Tae Lee**

Supporting Information

Towards Ideal Low-Frequency Noise in Monolayer CVD MoS_2 FETs: Influence of van der Waals Junctions and Sulfur Vacancy Management

Wonjun Shin[†,*], *Junsung Byeon*[†], *Ryun-Han Koo, Jungmoon Lim, Jung Hyeon Kang, A-Rang Jang, Jong-Ho Lee, Jae-Joon Kim, SeungNam Cha*[*], *Sangyeon Pak*[*], *Sung-Tae Lee*[*]

Dr. W. Shin, R. H. Koo, Prof. J. H. Lee, Prof. J. J. Kim

Inter-University Semiconductor Research Center, Department of Electrical and Computer Engineering, Seoul National University, Seoul 08826, Republic of Korea
Email: swj0107@snu.ac.kr
Dr. W. Shin
Research Laboratory of Electronics, Massachusetts Institute of Technology, Cambridge, MA, USA.
J. Byeon, J. Lim, Prof. S. Cha
Department of Physics, Sungkyunkwan University, Suwon, Gyeonggi-do 16419, Republic of Korea
Email: chasn@skku.edu
J. H. Kang, Prof. A. R. Jang
Division of Electrical, Electronic and Control Engineering, Kongju National University, Cheonan, 31080 Republic of Korea
Prof. J. H. Lee
Ministry of Science and ICT, Sejong 30109, Korea
Prof. S. Pak, Prof. S. T. Lee
School of Electronic and Electrical Engineering, Hongik University, Seoul 04066, Republic of Korea
Email: spak@hongik.ac.kr, lst777@hongik.ac.kr

[†]W. Shin and J. Byeon contributed equally to this work.

Supplementary figures

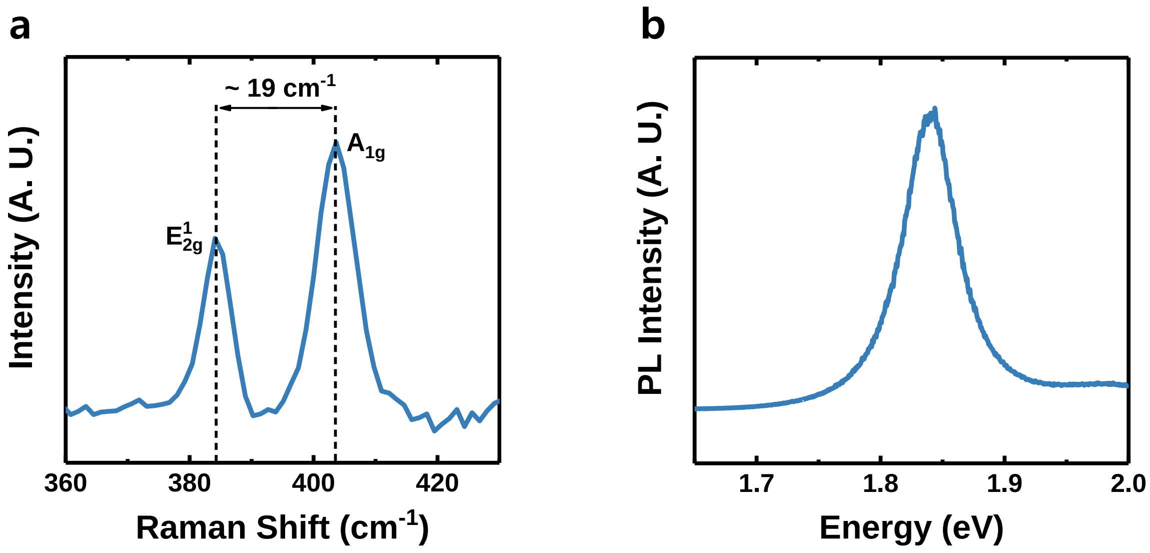

Figure S1. (a) Raman and (b) PL spectrum of CVD-growth monolayer MoS_2

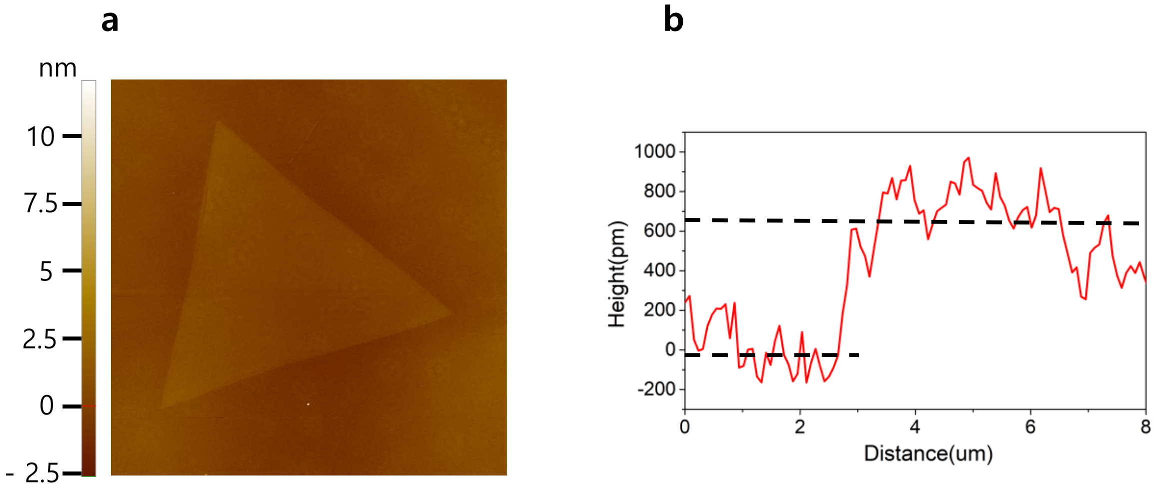

Figure S2. (a) AFM mapping image of monolayered MoS_2. (b) Height profile extracted from the mapping image.

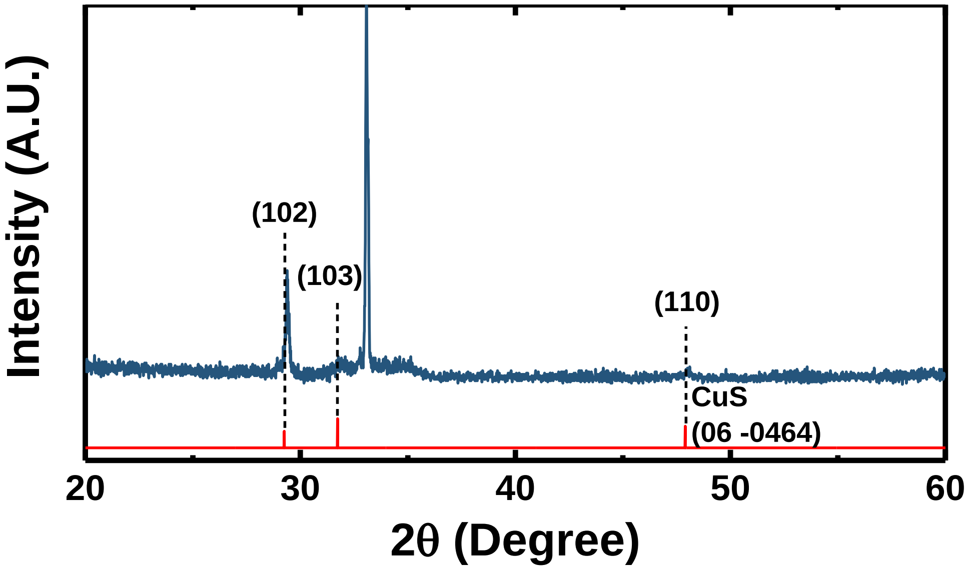

Figure S3. XRD spectra of CuS electrode with standard XRD spectra of covellite CuS structure (JCPDS No. 06-0464).

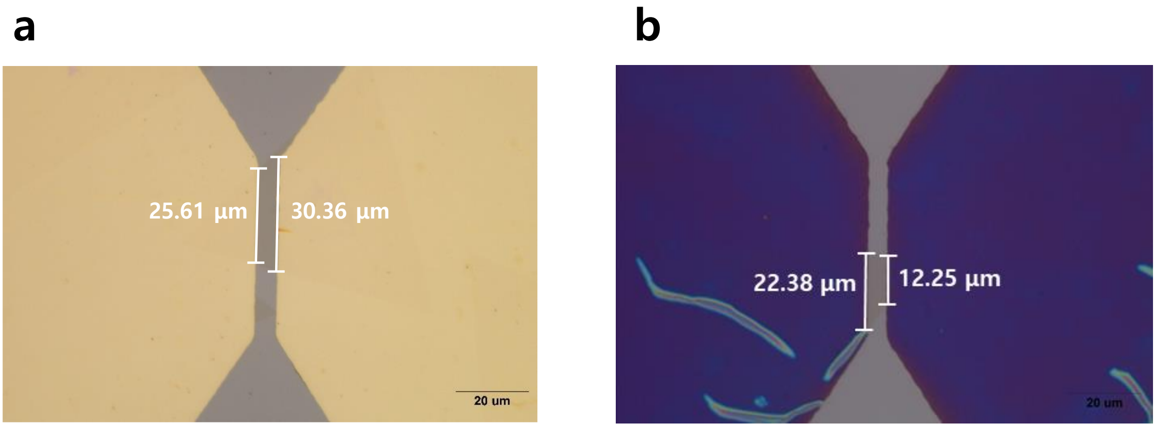

Figure S4. Optical images of the fabricated MoS_2 FETs with (a) Au and (b) CuS electrodes, respectively.

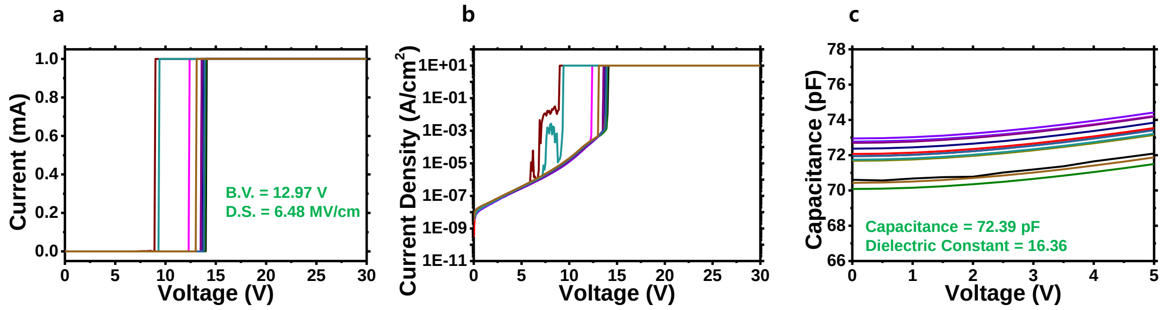

Figure S5. (a) Current, (b) current density, and (c) capacitance versus voltage of the HfO_2 film used as gate dielectric of the MoS_2 FETs.

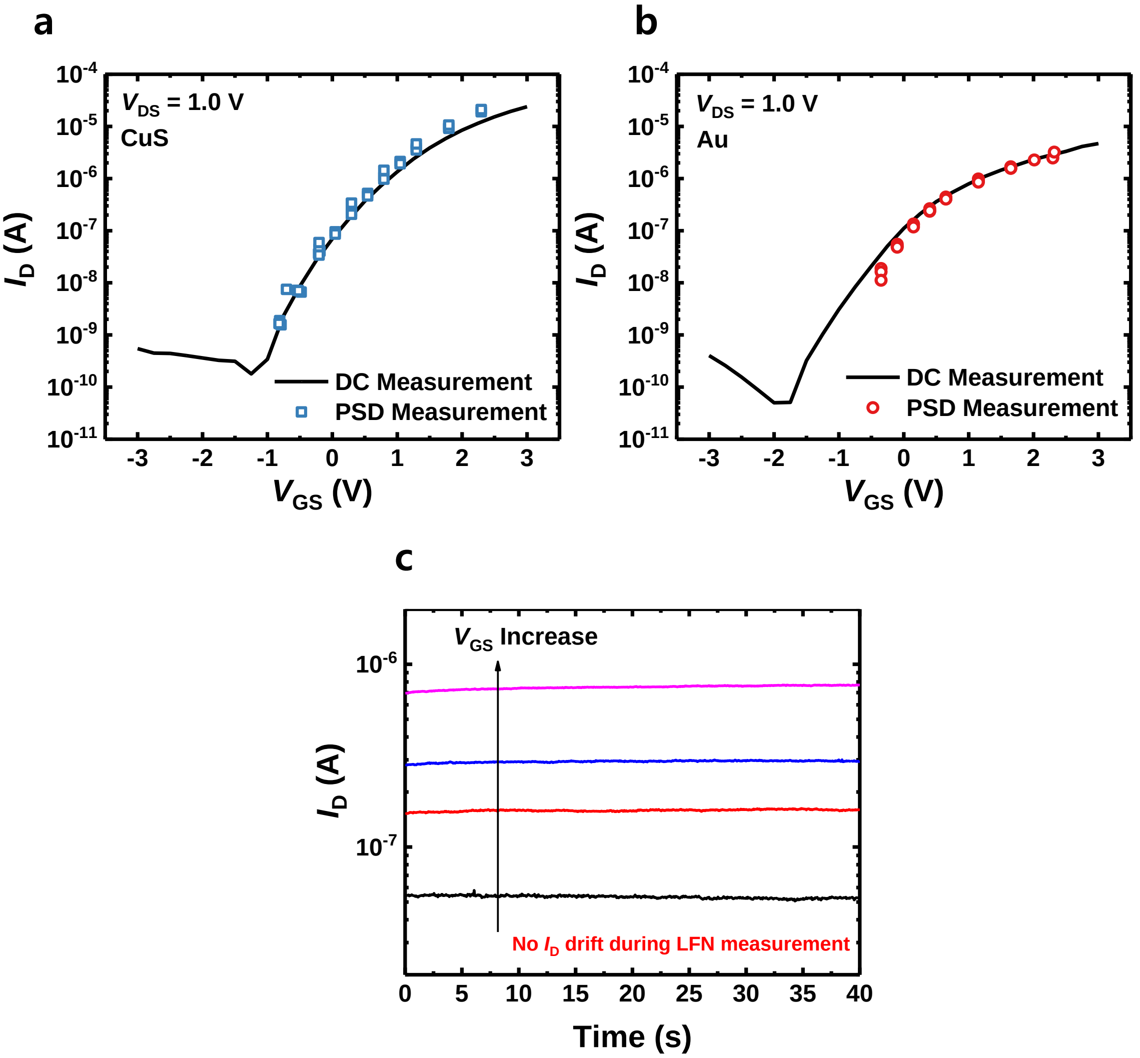

Figure S6. DC measured I_D-V_{GS} characteristics of the device and PSD measurement results of the FETs with (a) CuS and (b) Au electrodes, respectively. (c) Transisent I_D measurement results of the FETs during the PSD measurement.

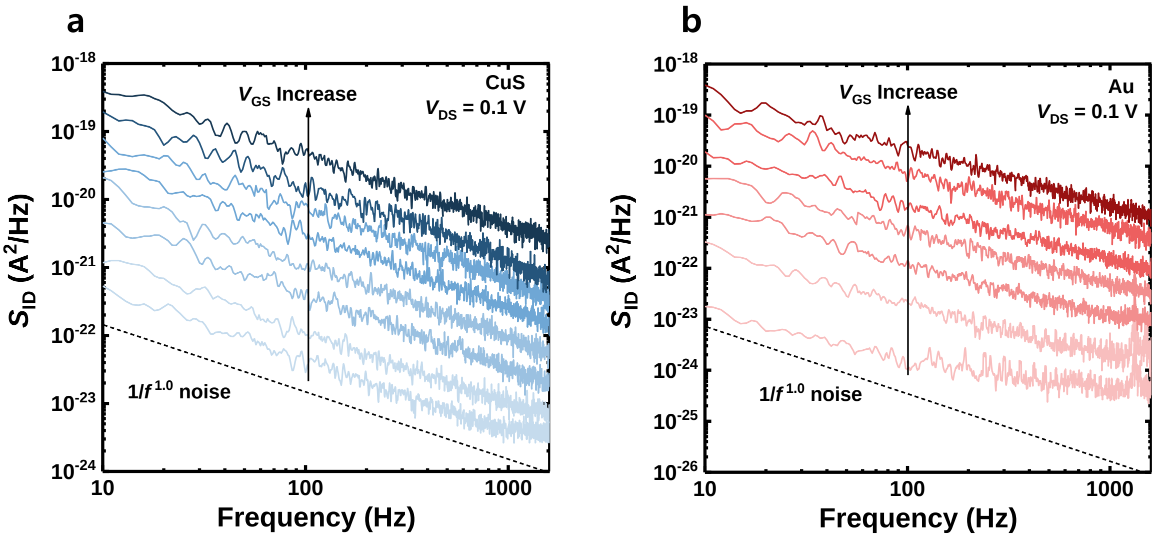

Figure S7. S_{ID} versus frequency for the MoS_2 FETs with (a) CuS and (b) Au electrodes, respectively. The V_{DS} is set at 0.1 V during the measurement, and the V_{GS} is increased to investigate the LFN behavior in different operating regions.

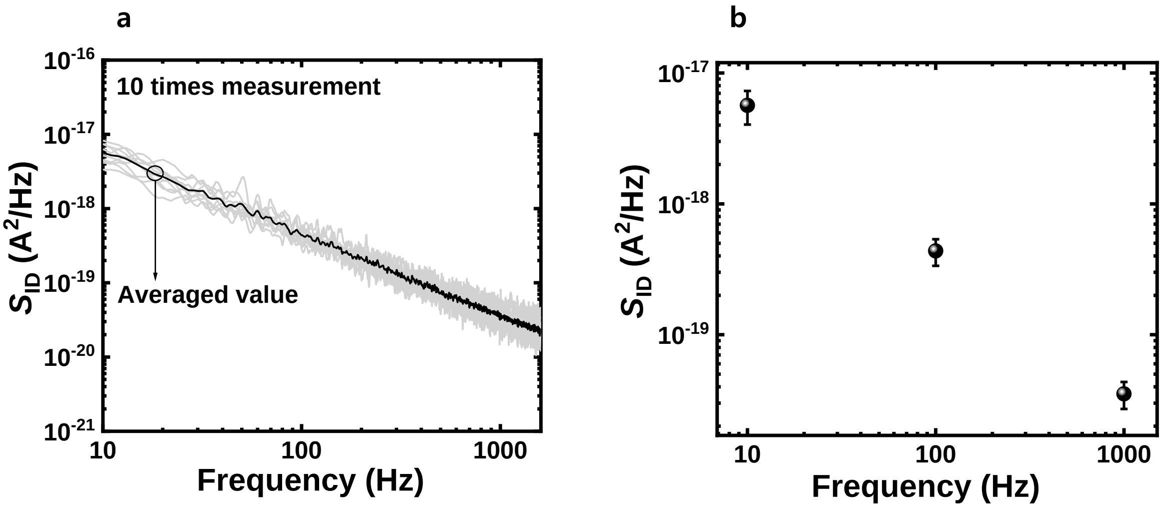

Figure S8. (a) Ten times of repeated measurement results of the PSD. The bold black line represents the average value. (b) statistics of the S_{ID} value sampled at 10, 10^2, and 10^3 Hz.

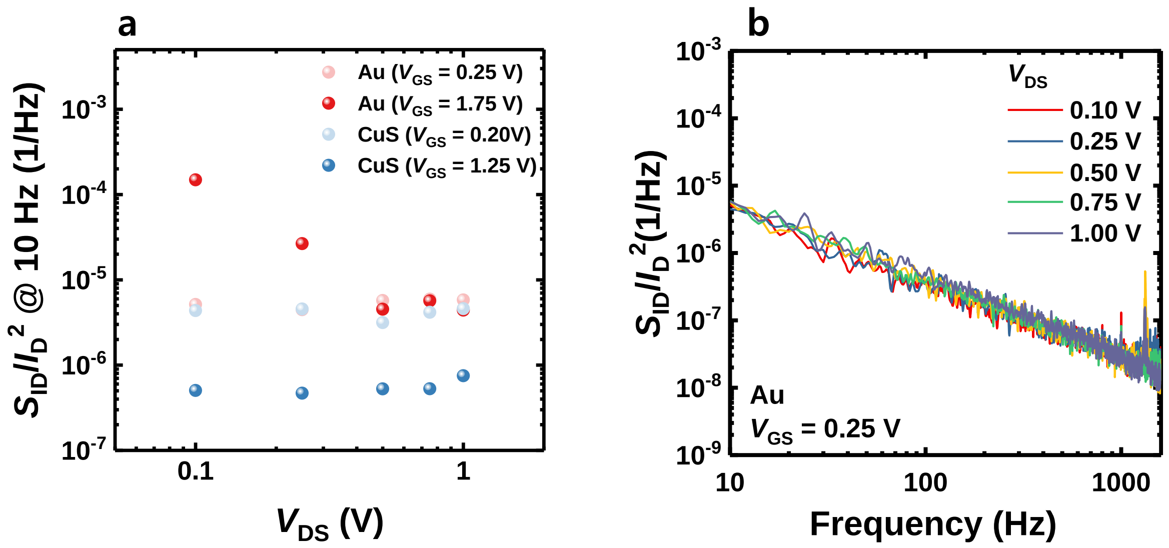

Figure S9. (a) Log-log plot of the S_{ID}/I_D^2 values sampled at 10 Hz versus V_{DS} of FETs with CuS and Au electrodes. (b) S_{ID}/I_D^2 of the FETs with Au electrode measured at different V_{DS} values.

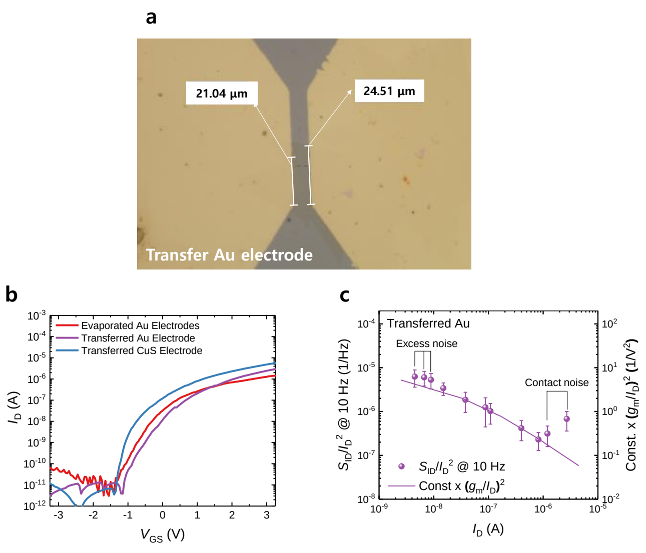

Fig. S10. (a) Optical images of the fabricated MoS_2 FET with transferred Au electrode. (b) Transfer characteristics of the MoS_2 FET with transferred Au electrode and other FETs in the manuscript. (c) S_{ID}/I_D^2 and $(g_m/I_D)^2$ versus I_D of the MoS_2 FET with transferred Au electrode.

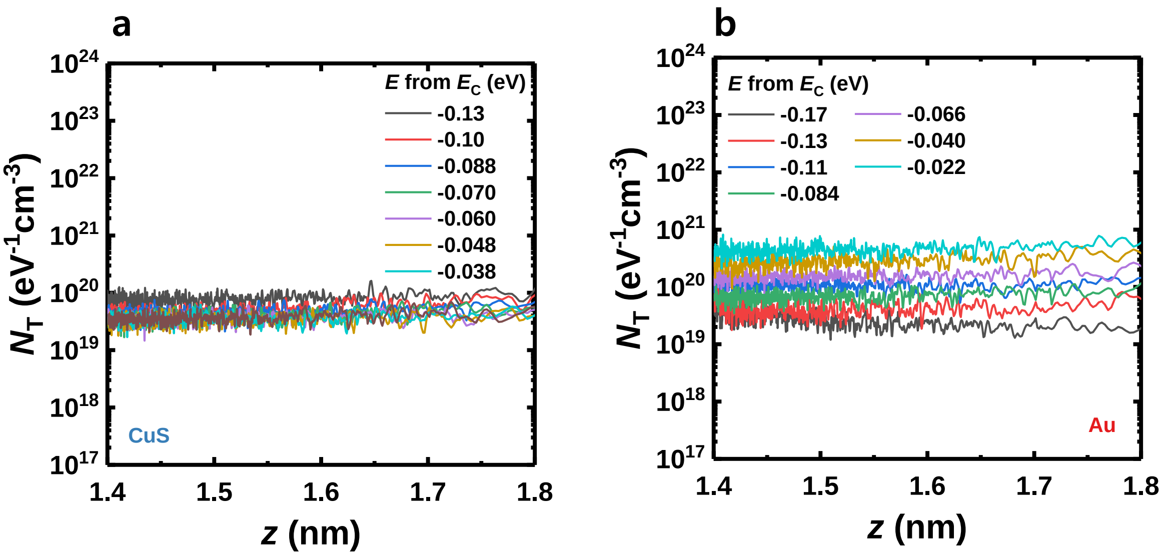

Figure S11. N_T as a function of z for various energy values of the FETs with (d) CuS and (e) Au electrodes, respectively.

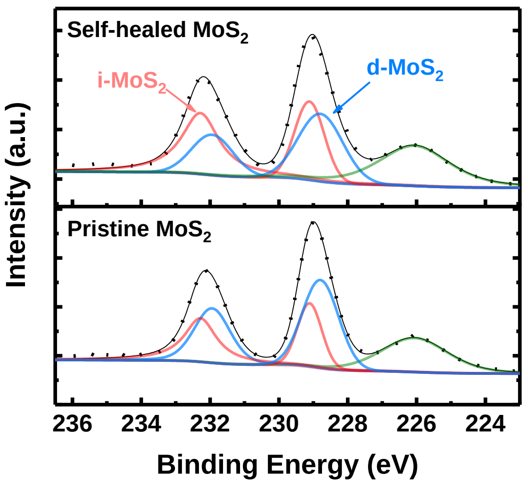

Figure S12. XPS spectra of Mo 3d peak in pristine MoS_2 and self-healed MoS_2 with CuS electrode.

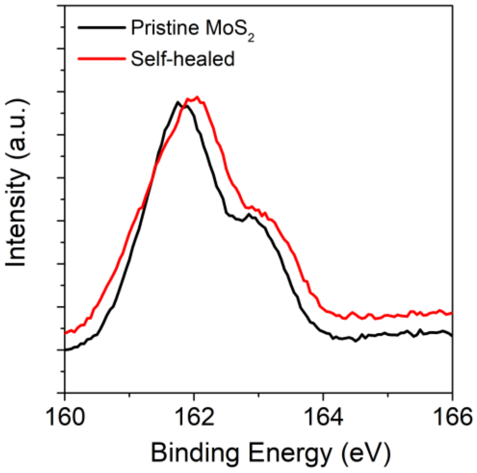

Figure S13. XPS measurement of S 2p orbital of MoS_2 and self-healed MoS_2.

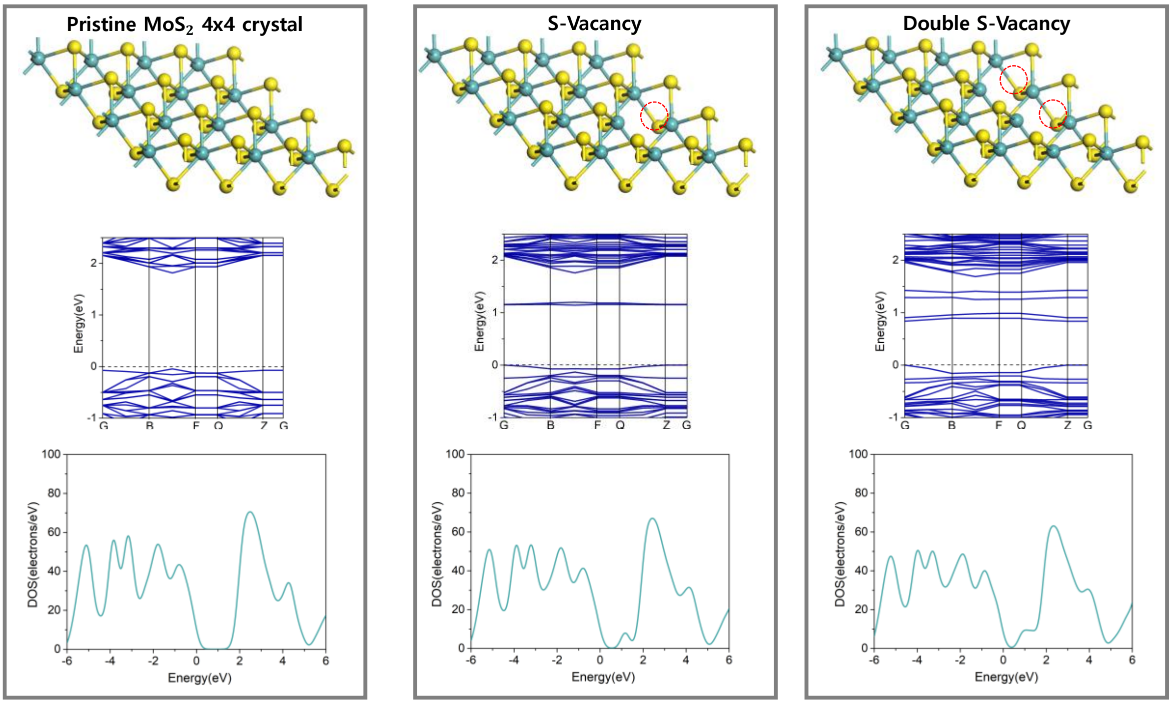

Figure S14. DFT calculation for 4x4 MoS_2 crystal. The band structure and density of states was calculated for the perfect MoS_2 crystal, MoS_2 crystal with sulfur vacancy (S-vacancy), and MoS_2 crystal with double S-vacancy.

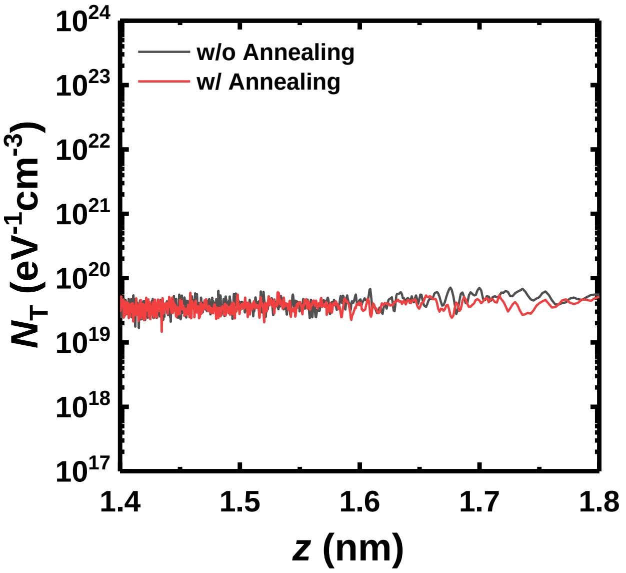

Figure S15. N_T of the MoS_2 FETs with CuS electrodes with and without thermal annealing.

Supplementary Table

Table S1. V_{th}, μ_{eff}, and N_{it} of the MoS_2 FETs with Au electrode, CuS electrode without and with thermal annealing.

	Au electrode	CuS electrode w/o annealing	Cus electrode w/ annealing
V_{th}	-0.29 V	-0.76 V	-0.84 V
μ_{eff}	4.41 $cm^2V^{-1}s^{-1}$	36.19 $cm^2V^{-1}s^{-1}$	84.24 $cm^2V^{-1}s^{-1}$
N_{it}	$9.82 \times 10^{10}/cm^2$	$3.41 \times 10^{10}/cm^2$	$2.47 \times 10^{10}/cm^2$

Supplementary Note 1

To establish the correlation between E_F-E_C and V_{GS}, the I_D-V_{GS} relation needs to be transformed into the I_D-energy relation. In the case of 2D FETs, I_D, which it the function of V_{GS}, is expressed as

$$I_{\mathrm{D}}(V_{\mathrm{GS}}) = AqN_{\mathrm{ch}}(V_{\mathrm{GS}})\mu_{\mathrm{eff}}E \quad (1)$$

where A represents the conducting channel area, q is the electron charge, $N_{ch}(V_{GS})$ denotes the electron concentration in the channel, μ_{eff} is the effective mobility, and E is the lateral electric field. The $N_{ch}(V_{GS})$ can be expressed as

$$N_{\mathrm{ch}}(V_{\mathrm{GS}}) = N_{\mathrm{C}}\exp(\frac{E_{\mathrm{F}}(V_{\mathrm{GS}})-E_{\mathrm{C}}}{kT}) \quad (2)$$

where N_C is the DOS in the conduction band minimun. Substituting (2) into (1),the I_D can be expressed as

$$I_{\mathrm{D}}(V_{\mathrm{GS}}) = AqN_{\mathrm{C}}\mu_{\mathrm{eff}}E\exp(\frac{E_{\mathrm{F}}(V_{\mathrm{GS}})-E_{\mathrm{C}}}{kT}) \quad (3).$$

Thus, the I_D in the conduction band minimum ($I_{D,CBM}$) is obtained as

$$I_{\mathrm{D,CBM}} = qWT_{\mathrm{ch}}N_{\mathrm{C}}\mu_{\mathrm{eff}}\frac{V_{\mathrm{DS}}}{L} \quad (4)$$

where W, L, and T_{ch} are the width, length, and thickness of the channel, respectively. By combining (3) and (4) and taking a log for the I_D, the relationship between the I_D-energy can be extracted as follows

$$\ln\left(I_{\mathrm{D}}(V_{GS})\right) = \ln\left(I_{\mathrm{D,CBM}}\right) + \frac{E_{\mathrm{F}}(V_{\mathrm{GS}})-E_{\mathrm{C}}}{kT} \quad (5).$$

Thus, the following equation can be obtained

$$\frac{dln(I_{\mathrm{D}}(V_{\mathrm{GS}}))}{dV_{\mathrm{GS}}} = \frac{1}{kT}\frac{dE_F(V_{\mathrm{GS}})}{dV_{\mathrm{GS}}} = \frac{g_{\mathrm{m}}(V_{\mathrm{GS}})}{I_{\mathrm{D}}(V_{\mathrm{GS}})} \quad (6).$$

By integrating (6) with respect to V_{GS}, the relationship between the E_F and V_{GS} can be extracted as follows

$$E_{\mathrm{F}}(V_{\mathrm{GS}}) - E_{\mathrm{REF}} = kT\int_{V_{\mathrm{th}}+V_{DS}}^{V_{\mathrm{GS}}} \frac{g_{\mathrm{m}}(V_{\mathrm{GS}})}{I_{\mathrm{D}}(V_{\mathrm{GS}})}dV_{\mathrm{GS}} \quad (7)$$

where E_{REF} is defined as E_F ($V_{GS} = V_{th} + V_{DS}$). The relationship between the ln(I_D) and E_F has the slope of $1/kT$ in the plot, demonstrating the validity of (7). By calculating the $I_{D,CBM}$ using (4) and extracting V_{CBM} using $I_{D,CBM}$, the relationship between the E_F-E_C versus V_{GS} can be extracted, as shown in Figure 4b. We have added relevant information in the supplementary note. Please refer to the revised manuscript to find the modifications.
